# microRNA-130a-5p suppresses myocardial ischemia reperfusion injury by downregulating the HMGB2/NF-κB axis

**DOI:** 10.1186/s12872-020-01742-4

**Published:** 2021-03-03

**Authors:** Yong Li, Hongbo Zhang, Zhanhu Li, Xiaoju Yan, Yuan Li, Shuai Liu

**Affiliations:** Department of Cardiology, Harrision International Peace Hospital, No. 180 Renmin East Road, Hengshui, 053000 Hebei People’s Republic of China

**Keywords:** Myocardium ischemia reperfusion injury, microRNA-130a-5p, HMGB2, NF-κb pathway, Oxidative stress, Mitochondrial disorder

## Abstract

**Background:**

Myocardial ischemia reperfusion injury (MIRI) is defined as tissue injury in the pathological process of progressive aggravation in ischemic myocardium after the occurrence of acute coronary artery occlusion. Research has documented the involvement of microRNAs (miRs) in MIRI. However, there is obscure information about the role of miR-130a-5p in MIRI. Herein, this study aims to investigate the effect of miR-130a-5p on MIRI.

**Methods:**

MIRI mouse models were established. Then, the cardiac function and hemodynamics were detected using ultrasonography and multiconductive physiological recorder. Functional assays in miR-130a-5p were adopted to test the degrees of oxidative stress, mitochondrial functions, inflammation and apoptosis. Hematoxylin and eosin (HE) staining was performed to validate the myocardial injury in mice. Reverse transcription quantitative polymerase chain reaction (RT-qPCR) was employed to assess the expression patterns of miR-130a-5p, high mobility group box (HMGB)2 and NF-κB. Then, dual-luciferase reporter gene assay was performed to elucidate the targeting relation between miR-130a-5p and HMGB2.

**Results:**

Disrupted structural arrangement in MIRI mouse models was evident from HE staining. RT-qPCR revealed that overexpressed miR-130a-5p alleviated MIRI, MIRI-induced oxidative stress and mitochondrial disorder in the mice. Next, the targeting relation between miR-130a-5p and HMGB2 was ascertained. Overexpressed HMGB2 annulled the protective effects of miR-130a-5p in MIRI mice. Additionally, miR-130a-5p targets HMGB2 to downregulate the nuclear factor kappa-B (NF-κB) axis, mitigating the inflammatory injury induced by MIRI.

**Conclusion:**

Our study demonstrated that miR-130a-5p suppresses MIRI by down-regulating the HMGB2/NF-κB axis. This investigation may provide novel insights for development of MIRI treatments.

## Background

Over production of free radicals and reactive oxygen species (ROS) has been identified as the principal cause for the pathogenesis of myocardial ischemia reperfusion injury (MIRI) [1]. Reperfusion after ischemia exacerbates the severity of ischemic injury and serves as a vital cause for about a half of myocardial infarction (MI) size [2]. The cascade of various inflammatory factors induces secondary injury, exacerbating IR injury [3]. Oxidative stress consequent of MIRI in an open-heart surgery or MI aggrandizes the accumulative reactive aldehydes, thus precipitating to severe heart injury [4]. Additionally, the systematic mitochondria are an effective method to appease MIRI, accounting that the ROS-mediated mitochondrial disorder can accentuate the risk of higher susceptibility to MI, heart dysfunction and incidence of heart arrhythmia [5]. However, due to the lack of concrete evidence to thoroughly avoid MIRI [6], novel therapeutic strategies are imperative for the prevention of MIRI. Therefore, our study was designed to investigate the roles of different pathological mechanisms in MIRI, in order to develop novel intervention strategies.

microRNAs (miRs) are acknowledged for their involvement in MIRI by serving as modulators of pivotal signaling factors [7]. During the various MIRI states, miRs are differentially expressed to influence the MIRI biological developments [8]. Additionally, the miR-130a ectopic expression reduces ROS production in patients with oxygen–glucose deprivation reperfusion (OGDR) [9], thereby exhibiting its protective properties in patients affected with diseases associated to ischemic reperfusion. Furthermore, Lu et al. [10] have elicited that miR-130a aids in reducing MI through remodeling and promoting cardiac functions. In acute ischemic stroke tissues, miR-130a is conducive in reducing the susceptibility and severity to disease and decreasing the levels of inflammatory factors [11]. Furthermore, miR-130a-3p could accentuate the angiogenesis in MI as a protective measure for the ischemic heart [12]. Moreover, miR-130a-5p is downregulated in human definitive endodermal progenitor cells, which principally serves as a target for mitochondria, denoting a positive relation between miR-130a-5p and mitochondrial structure and function remodeling [13]. HMGB2 (high mobility group box 2) is regarded as a trivial factor in severe ischemic injury of MI through augmenting the inflammatory response, cell apoptosis and autophagosome clearance damage by facilitating ROS [14]. NF-κB serves as an integrated transcriptional factor in cardiovascular disease [15]. NF-κB is highly expressed in lung IRI rats [16]. HMGB2^−/−^ mouse cells are defective in type-I interferon and inflammatory cytokine, showing impaired activation of NF-κB [17]. From the aforementioned literature, it is hypothesized that miR-130a-5p/HMGB2/NF-κB can be a viable target in MIRI treatment. Thus, our experiments were performed to substantiate the hypothesis.

## Methods

### Animal grouping

A total of sixty healthy C57BL/6 male mice (6–8-week-old, 20–25 g) (Guangzhou General Pharmaceutical Research Institute Co., Ltd., Guangzhou, Guangdong, China, SYXK (Guangdong) 2018-0003) raised in standard conditions were numbered with body weight as a parameter and randomly allocated into the sham group, MIRI group, agomir negative control (NC) group, miR-130a-5p agomir group, miR-130a-5p agomir + pLVX-Puro group and miR-130a-5p agomir + pLVX-Puro-HMGB group, with 10 mice in each group. Mice in the sham group were threaded in the left anterior descending coronary artery without ligation. Mice in the MIRI group were anesthetized using an intraperitoneal injection of 4% pentobarbital sodium (40 mg/kg), after which their limbs were connected to the BL-420F physiological monitor system (Techman Science and Technology Co., Ltd., Chengdu, Sichuan, China) for subsequent electrocardiography, and their chests were dissected on the fourth rib, and ligated for 30 min via the left cardiac anterior descending coronary artery and then underwent a 120-min reperfusion. Correspondingly, the mice in the remaining 4 groups 24 h before MIR were subjected to an intramyocardial injection of agomir NC (Guangzhou RiboBio Co., Ltd, Guangzhou, Guangdong China), miR-130a-5p agomir (50 µL, 300 nmol/kg) [18] (RiboBio), miR-130a-5p agomir + pLVX-Puro (1 × 10^7^ pfu) (Gaining Biotechnology Co., Ltd., Shanghai, China) and miR-130a-5p agomir + pLVX-Puro-HMGB2 (1 × 10^7^ pfu) (Gaining) respectively.

### Detection of cardiac function and hemodynamics in mice

After reperfusion, the Philips Sonos 5500 color Doppler ultrasonography (Philips, Andover, MA, USA) was utilized to determine the left ventricle (LV) ejection fraction (EF) and LV fraction shortening (FS). After the echocardiography, numerous self-made catheters with heparin saline were inserted via the right common carotid artery of mice into the LV. Then, the systolic pressure (SP), end-diastolic pressure (EDP) and the maximal rate of rise and fall (± dp/dtmax) of the LV were analyzed and documented using a multi-conductive physiological recorder (Chengdu Medical Instruments Co., Ltd., Chengdu, Sichuan, China).

### Sample collection

After the hemodynamics detection, the mice were euthanized using an intraperitoneal injection with 40 mg/kg pentobarbital sodium, and 1 mL blood samples collected from each mouse by cardiac puncture were placed for 10 min and then centrifuged at 3000 g for 10 min. The 200 μL serum was extracted and preserved at − 80 °C for subsequent experimentation. After collecting the blood samples, the mice were sacrificed by an intraperitoneal injection of excessive pentobarbital sodium, after which the myocardial tissues were harvested. Among each group, 4 samples of myocardial tissues were adopted to prepare tissue homogenate and the remaining 6 samples were fixed using 4% paraformaldehyde for 24 h and were methodically prepared into paraffin-sections for histological staining.

### Enzyme-linked immunosorbent assay (ELISA)

The serum was diluted, and the levels of creatine kinase (CK), T Cardiac troponin T (cTnT), lactate dehydrogenase (LDH), superoxide dismutase (SOD) and malondialdehyde (MDA) in the mouse serum (20 μL/well) were analyzed in duplicates in strict accordance with the provided instructions (NanJing JianCheng Bioengineering Institute, Nanjing, Jiangsu, China). The levels of tumor necrosis factor-α (TNF-α), interleukin (IL)-6 and IL-1β were evaluated using the enzyme-linked immunosorbent assay kits (Shanghai Enzyme-linked Biotechnology Co., Ltd., Shanghai, China).

### Measurement of mitochondrial functions

With the addition of the mitochondrial separation reagent (Beyotime Biotechnology Co., Ltd., Shanghai, China), the tissue homogenate was centrifuged at 600*g* at 4 °C for 5 min, and the supernatant was extracted and centrifuged at 1100*g* at 4 °C for 10 min, followed by supernatant removal. Next, the mitochondria were resuspended using the mitochondrial storage fluid for the detection of various mitochondrial function-related indices. JC-1 kit (Beyotime) was employed to assess the mitochondria membrane potential (MMP). Additionally, the levels of adenosine triphosphate (ATP), ROS and calcium ion were measured based on the provided instructions of the ATP kit (Shanghai Enzyme-linked Biotechnology), ROS kit (Shanghai Enzyme-linked Biotechnology) and the calcium ion detection kit (Sigma-Aldrich, Merck KGaA, Darmstadt, Germany) respectively.

### Hematoxylin and eosin (HE) staining

The regularly dewaxed and dehydrated paraffin-sections were stained using hematoxylin (Beijing Solarbio Science and Technology Co., Ltd., Beijing, China) for 3 min and then rinsed. Subsequently, these sections were differentiated using 1% hydrochloric alcohol for 15 s. After a regimen of 2-min staining using eosin (Solarbio), the sections were observed under an optical microscope (Olympus Optical Co., Ltd., Tokyo, Japan).

### Terminal deoxynucleotidyl transferase (TdT)–mediated dUTP nick-end labeling (TUNEL) staining

After the paraffin-sections were regularly dewaxed and dehydrated, they were analyzed in strict accordance with the provided instructions of the TUNEL apoptosis detection kit (Shanghai Yeason Bio Technologies Co., Ltd., Shanghai, China). Next, the nuclei were stained with 4′,6-diamidino-2-phenylindole (Beyotime) for observation of the TUNEL-positive cells under a fluorescence microscope (Olympus).

### Reverse transcription-quantitative polymerase chain reaction (RT-qPCR)

The total RNA content from the tissue homogenate of each group was extracted using a TRIzol kit (Beyotime) for assessment of RNA concentration and purity. Then, the extracted RNA content was reverse transcribed into cDNA using the Rever Tra Ace qPCR RT Master Mix kit (Toyobo Co., Ltd., Tokyo, Japan). SYBR Premix Ex Taq™ II (Takara, Dalian, China) was applied to perform fluorescent qPCR. The 2^−ΔΔCt^ method was adopted to calculate the relative expression of the genes with glyceraldehyde-3-phosphate dehydrogenase (GAPDH) as the internal reference (Table 1).

**Table 1:** Primers sequence

Gene	Primers (5′–3′)
miR-130a-5p	Forward: 5′-CCAGGGCTTTTCAAAAATGA-3′
	Reverse: 5′-CCGATCCAATCTGTTCTGGT-3′
U6	Forward primer: 5′-GTGCTCGCTTCGGCAGCA-3′
	Reverse primer: 5′-CAAAATATGGAACGCTTC-3′
HMGB2	Forward primer: 5′-GGGAAGAGCACAAGAAGAAAC-3′
	Reverse primer: 5′-AAACAGGAAGAAGGCAGATGG-3′
GAPDH	Forward primer: 5′-GTCAACGGATTTGGTCTGTATT-3′
	Reverse primer:5′-AGTCTTCTGGGTGGCAGTGAT-3′

*miR* microRNA, *HMGB* high mobility group protein, *GAPDH* glyceraldehyde-3-phosphate dehydrogenase

### Western blot analysis

The total protein content from the tissue homogenate of each group was extracted for measurement of protein concentration. Next, the protein content was subjected to sodium dodecyl sulfate polyacrylamide gel electrophoresis (Beyotime) and then transferred onto polyvinylidene fluoride membranes. Then, the membranes were sealed using 5% skim milk powder for 1 h and cultured with the following primary antibodies (all from Abcam Inc., Cambridge, MA, USA): B-cell lymphoma-2 (Bcl-2) (ab59348, 1:1000), Bcl2-associated X (Bax) (ab182733, 1:2000), Cytochrome C (Cyt-c) (ab216971, 1:1000), apoptosis inducing factor (AIF) (ab32516, 1:1000), HMGB2 (ab124670, 1:10,000), p65 (ab16502, 0.5 μg/mL), inhibitor of κB (IκB)α (ab109300, 1:1000), p-p65 (ab86299, 1:2000) and p-IκBα (ab133478, 1:10,000) at 4 °C overnight. Next, the membranes were cultivated using the horseradish peroxidase labeled goat anti-rabbit immunoglobulin G (IgG) antibody (ab32152, 1:2000) or the anti-mouse IgG antibody (ab205719, 1:2000) at room temperature for 1 h and then exposed for visualization and observation. GAPDH (ab8245, 1:1000) served as the internal reference, and the ratio of the gray value of the target protein band to GAPDH was used as the relative protein expression.

### Dual-luciferase reporter gene assay

TargetScan predicted the presence of numerous binding sites between miR-130a-5p and HMGB2. The complementary binding sequence of miR-130a-5p and HMGB2 was amplified using PCR and cloned to the pmiR-GLO luciferase vector (Promega, Madison, WI, USA) in order to construct HMGB2 wild type (WT) plasmids and HMGB2 mutant type (MUT) plasmids. Then, the two kinds of plasmids were combined with mimic NC and miR-130a-5p mimic and then transfected into the 293 T cells with Lipofectamine™ 2000 (Invitrogen Inc., Carlsbad, CA, USA). The luciferase activity was evaluated 48 h later. All experiments were conducted 3 times independently.

### Statistical analysis

SPSS 21.0 (IBM Corp. Armonk, NY, USA) was employed for statistical data analysis. Kolmogorov–Smirnov test was employed to assess whether the data were in normal distribution. The results were shown in mean ± standard deviation. One-way analysis of variance (ANOVA) or two-way ANOVA was adopted for comparing among different groups, and the Tukey’s multiple comparisons test was adopted for pairwise comparisons after ANOVA. The *p* value was attained using a two-tailed test, where a value of *p* < 0.05 was indicative of a significant difference.

## Results

### Identification of MIRI mouse models

**Fig. 1:**
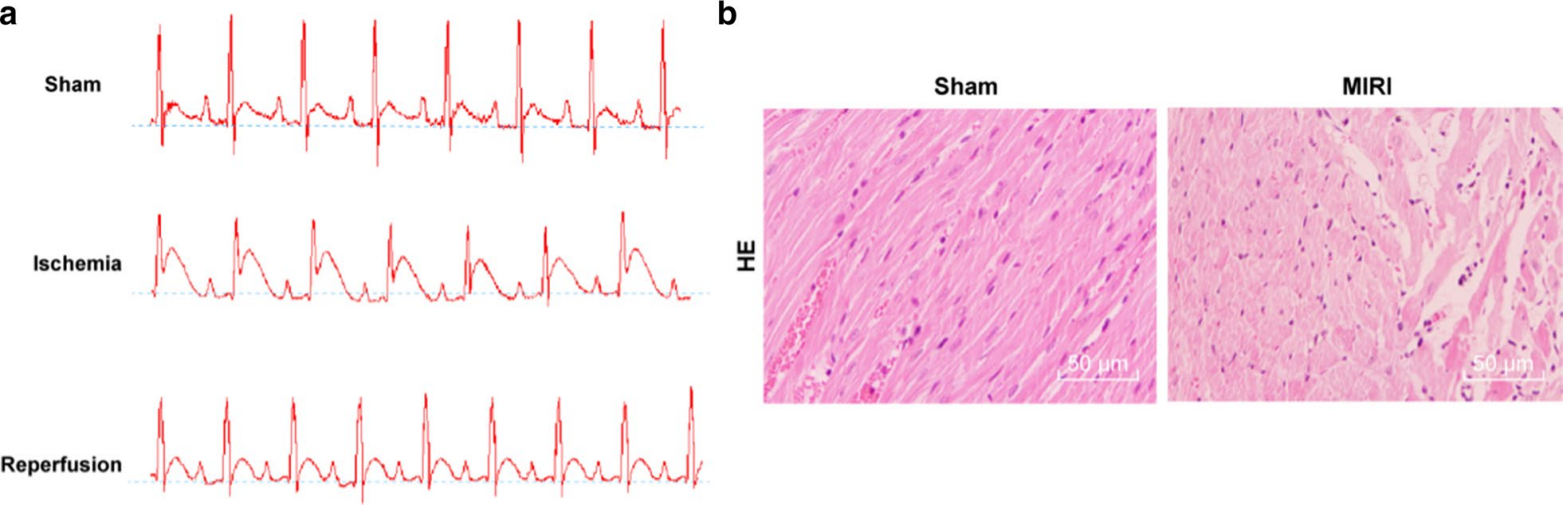
Identification of MIRI mouse models. **a** The electrocardiography of the MIRI mice, ischemia: 30 min, reperfusion: 120 min, n = 10. **b** HE staining revealed that the myocardial tissues were disrupted in the MIRI mice, × 200, n = 6. *MIRI* myocardial ischemia reperfusion injury, *HE* hematoxylin and eosin

**Fig. 2:**
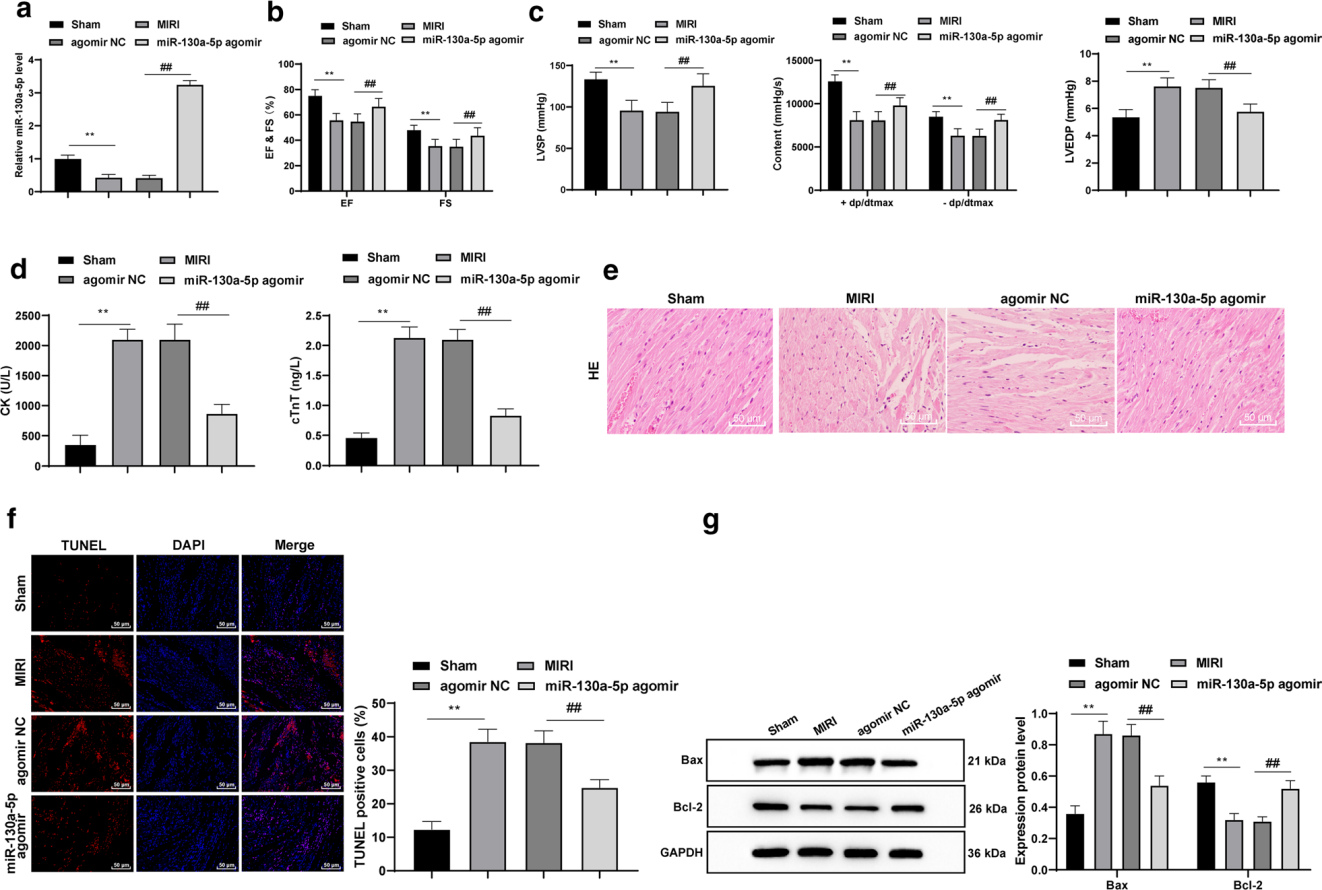
Overexpressed miR-130a-5p alleviates MIRI in mice. **a** RT-qPCR showed that the MIRI mice had a lower miR-130a-5p expression pattern, which was increased after overexpressing miR-130a-5p, n = 4. **b**, **c** The cardiac function and hemodynamics in mice were detected, n = 10. **d** ELISA results suggested notable increases in serum CK and cTnT levels from the MIRI mice, which were repressed by overexpressed miR-130a-5p, n = 10. **e** HE staining revealed that the disrupted myocardial tissues were improved with overexpressed miR-130a-5p, × 200, n = 6. **f** TUNEL assay revealed that the high apoptosis rates in cardiomyocyte in MIRI mice was declined by overexpressing miR-130a-5p, × 400, n = 6. **g** Western blot analysis demonstrated that the growth of Bax expression pattern and slowdown of Bcl-2 expression pattern in mice caused by MIRI were reversed by miR-130a-5p overexpression, n = 4. The images of **g** shown are cropped. Two-way ANOVA was used to determine data in panels **c**, **g**, and one-way ANOVA was employed to determine data in panels **a**, **c**, **d**, **f**. Tukey’s multiple comparisons test was applied for the post hoc test. **p < 0.01; ^##^*p* < 0.01. *miR* microRNA, *MIRI* myocardial ischemia reperfusion injury, *RT-qPCR* reverse transcription quantitative polymerase chain reaction, *CK* creatine kinase, *cTnT* T Cardiac troponin T, *HE* hematoxylin and eosin, *TUNEL* terminal deoxynucleotidyl transferase (TdT)-mediated dUTP nick end labeling, *Bcl2* B-cell lymphoma-2, *Bax* Bcl2-Associated X, *ANOVA* analysis of variance

In comparison with the sham group, the ST segment of mice in the MIRI group rose and peaked until 30 min after ischemia and regressed at the 120 min after reperfusion (Fig. 1a). Results of HE staining observed consistent cardiomyocyte structure with orderly and tight myocardial fiber arrangement in the sham-operated mice; while in the MIRI mice, the broken and light-stained myocardial fibers were disorderly arranged with an extending interim between each other (Fig. 2b).

### Overexpressed miR-130a-5p alleviates MIRI in mice

In comparison with the sham-operated mice, the MIRI mice presented with a lower miR-130a-5p expression pattern, which was increased after overexpressing miR-130a-5p (all *p* < 0.01) (Fig. 2a). In the MIRI group, the EF, FS, LVSP and ± dp/dtmax were all reduced and LVEDP was elevated; while overexpressed miR-130a-5p resulted in contradictory outcomes relative to those in the sham group (all *p* < 0.01) (Fig. 2b, c). Significant increases were observed in the CK and cTnT contents in serum from the MIRI mice, which were repressed by overexpressed miR-130a-5p (all *p* < 0.01) (Fig. 2d). According to the HE staining, MIRI in mice was alleviated with miR-130a-5p overexpression (Fig. 2e). TUNEL assay revealed a high apoptosis rate in the cardiomyocytes in MIRI mice, which was lowered by overexpressing miR-130a-5p (all *p* < 0.01) (Fig. 2f). Western blot analysis revealed that the increase of Bax expression and decrease of Bcl-2 expression in the mice under MIRI were reversed by miR-130a-5p overexpression (all *p* < 0.01) (Fig. 2g).

### Overexpressed miR-130a-5p alleviates MIRI-induced oxidative stress and mitochondrial disorder in mice

**Fig. 3:**
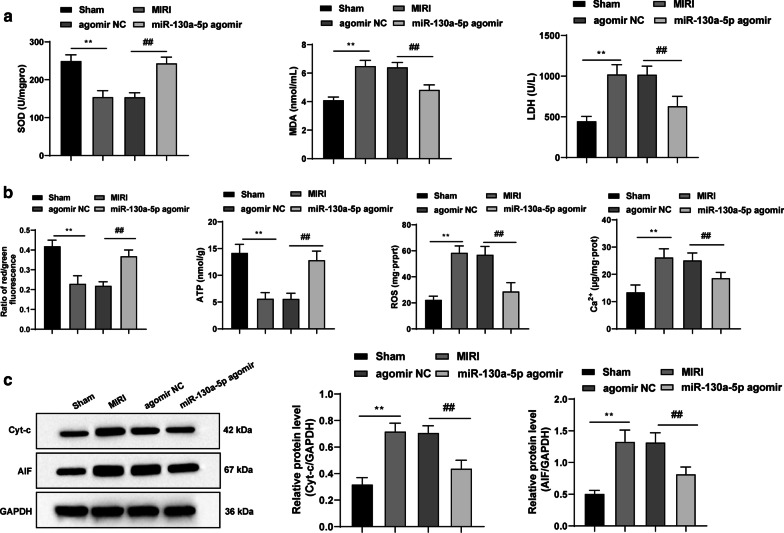
Overexpressed miR-130a-5p alleviates MIRI-induced oxidative stress and mitochondrial disorder in mice. **a** Serum levels of LDH, MDA and SOD measured using kits, n = 10. **b** MMP, ATP, ROS and Ca^2+^ expression were tested with kits, n = 4. **c** Western blot analysis showed that the elevated protein levels of Cyt-c and AIF in MIRI mice were discouraged by miR-130a-5p overexpression, n = 4. The images of **c** shown are cropped. One-way ANOVA and Tukey’s multiple comparisons test were applied to determine the statistical significance. **p < 0.01; ^##^*p* < 0.01. *miR* mircoRNA, *MIRI* myocardial ischemia reperfusion injury, *LDH* lactate dehydrogenase, *MDA* malondialdehyde, *SOD* superoxide dismutase, *MMP* mitochondrial membrane potential, *ATP* adenosine triphosphate, *ROS* reactive oxygen species, *Cyt-c* Cytochrome C, *AIF* apoptosis inducing factor, *ANOVA* analysis of variance

Relative to those in the sham group, the decreases in SOD, MMP and ATP as well as increases in LDH, MDA, ROS, Ca^2+^, Cyt-c and AIF protein levels were evident in the MIRI group, which were inverted by overexpressed miR-130a-5p (all *p* < 0.01) (Fig. 3a–c).

### miR-130a-5p targets HMGB2

**Fig. 4:**
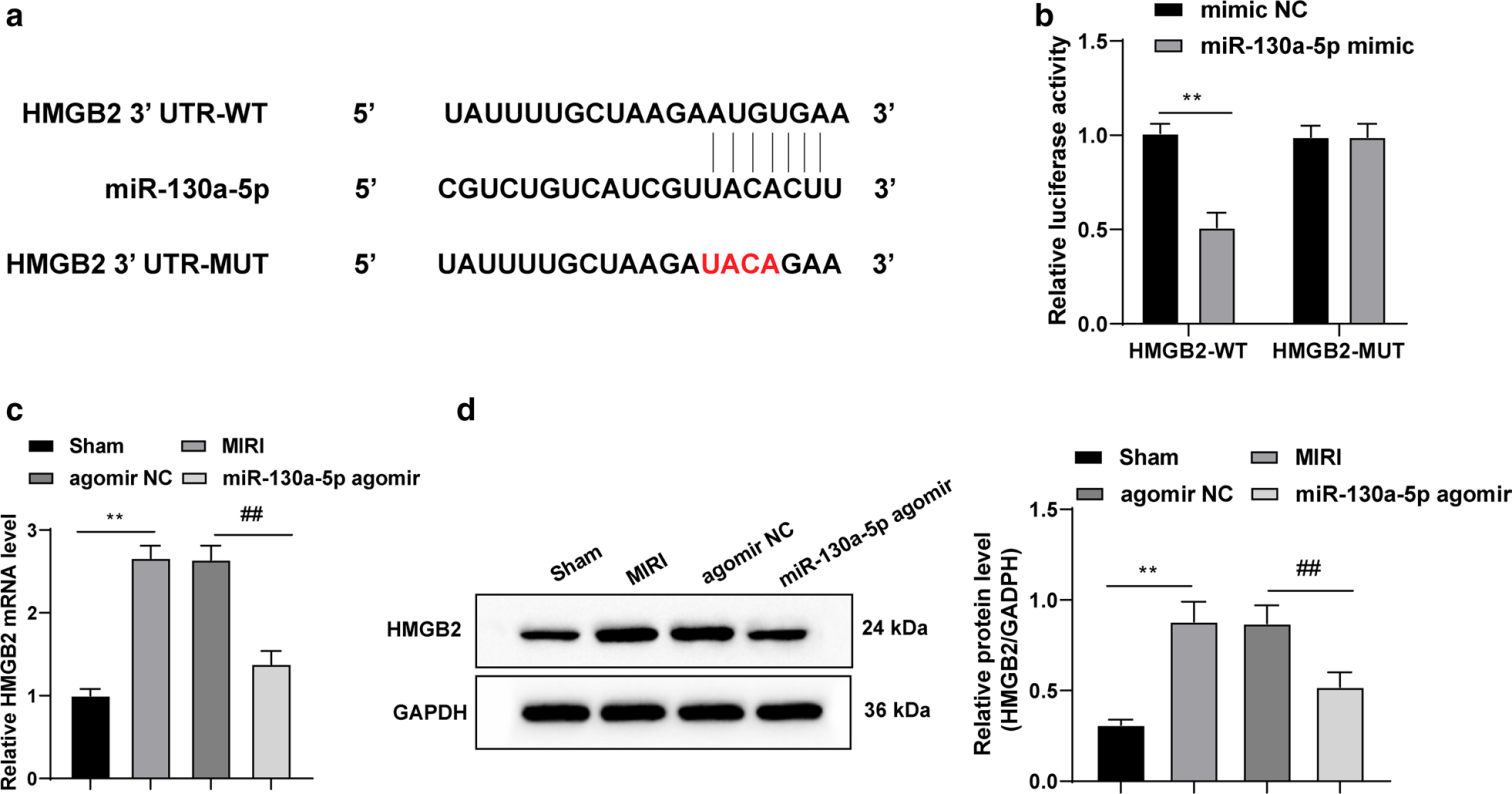
miR-130a-5p targets HMGB2. **a** TargetScan identified the binding site between miR-130a-5p and HMGB2 3′ untranslated region. **b** Dual-luciferase reporter gene assay verified the targeting relation between miR-130a-5p and HMGB2. **c**, **d** RT-qPCR and western blot analysis indicated that mRNA expression and protein level of HMGB2 in MIRI mice were both lowered by overexpressed miR-130a-5p, n = 4. The images of **d** shown are cropped. Two-way ANOVA was used to determine data in panel **b** and one-way ANOVA was employed to determine data in panels **c**, **d**. Tukey’s multiple comparisons test was applied for post hoc test. **p < 0.01; ^##^*p* < 0.01. *miR* microRNA, *HMGB* high mobility group box, *RT-qPCR* reverse transcription quantitative polymerase chain reaction, *MIRI* myocardial ischemia reperfusion injury, *ANOVA* analysis of variance

TargetScan identified the binding site between miR-130a-5p and HMGB2 3′ untranslated region (Fig. 4a). According to the results from the dual-luciferase reporter gene assay, in comparison with the control group, miR-130a-5p mimic significantly reduced the HMGB2-WT luciferase activity (both *p* < 0.01) (Fig. 4b). Additionally, the mRNA expression and protein level of HMGB2 in the MIRI mice were lowered by overexpressed miR-130a-5p (all *p* < 0.01) (Fig. 4c, d).

### Overexpressed HMGB2 reverses the protective effects of miR-130a-5p on MIRI mice

**Fig. 5:**
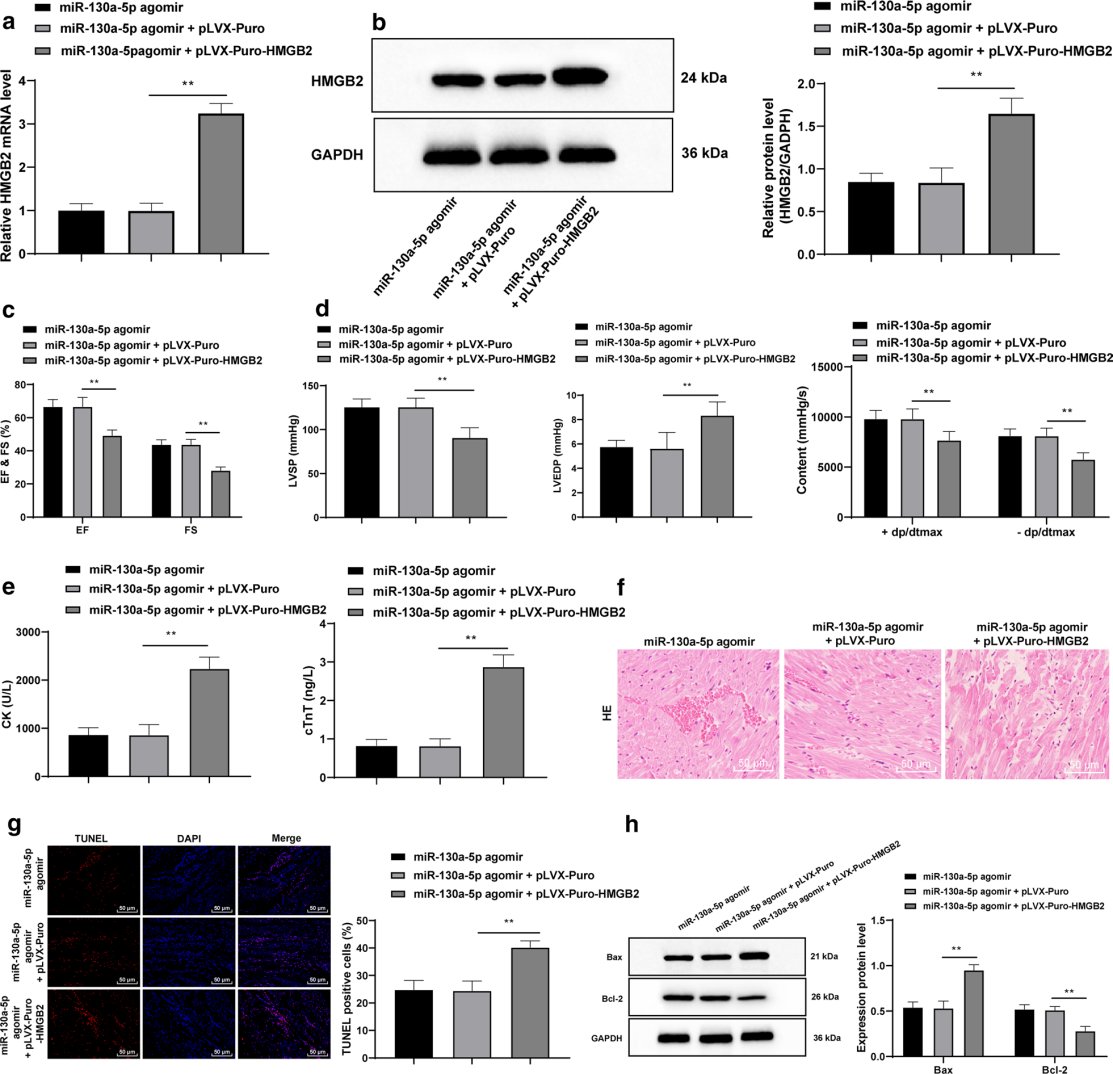
Overexpressed HMGB2 inverts the protective effects of miR-130a-5p on MIRI mice. **a**, **b** RT-qPCR and western blot analysis revealed that the mRNA expression and protein level of HMGB2 were evidently promoted when HMGB2 was up-regulated, n = 4. **c**, **d** EF, FS, LVSP and ± dp/dtmax were declined while LVEDP was enhanced when HMGB2 was up-regulated, n = 10. **e** CK and cTnT levels in serum were increased with up-regulated HMGB2 as detected using ELISA kits, n = 10. **f** myocardial injury was further deteriorated with up-regulated HMGB2 as detected by TUNEL assay, × 200, n = 6. **g** TUNEL assay suggested that the apoptosis rate of cardiomyocytes was enhanced with up-regulated HMGB2, × 400, n = 6. **h** Western blot analysis indicated that the Bax expression pattern was enhanced and the Bcl-2 expression pattern was reduced upon upregulated HMGB2, n = 4. The images of **b**, **h** shown are cropped. Two-way ANOVA was adopted to determine data in panels **c**, **d**, **h**, and one-way ANOVA was employed to determine data in panels **a**, **b**, **d**, **e**, **g**. Tukey’s multiple comparisons test was applied for post hoc test. **p < 0.01. *HMGB* high mobility group box, *miR* mircoRNA, *MIRI* myocardial ischemia reperfusion injury, *RT-qPCR* reverse transcription quantitative polymerase chain reaction, *EF* ejection fraction, *FS* fraction shortening, *LVSP* left ventricle systolic pressure, *± dp/dtmax* maximal rate of rise and fall, *LVEDP* left ventricle end-diastolic pressure, *CK* creatine kinase, *cTnT* T Cardiac troponin T, *TUNEL* terminal deoxynucleotidyl transferase (TdT)-mediated dUTP nick end labeling, *Bcl2* B-cell lymphoma-2, *Bax* Bcl2-Associated X, *ANOVA* analysis of variance

In comparison with the miR-130a-5p agomir group, when HMGB2 was upregulated in the context of miR-130a-5p overexpression, the mRNA expression and protein level of HMGB2 were evidently increased (all *p* < 0.01) (Fig. 5a, b); EF, FS, LVSP and ± dp/dtmax were lowered while the LVEDP was enhanced (all *p* < 0.01) (Fig. 5c, d); CK and cTnT levels in the serum were elevate (all *p* < 0.01) (Fig. 5e), myocardial injury was further deteriorated (Fig. 5f), the apoptosis rate of cardiomyocytes were enhanced (all *p* < 0.01) (Fig. 5g), Bax expression was elevated and Bcl-2 expression was reduced (all *p* < 0.01) (Fig. 5h).

### miR-130a-5p targets HMGB2 to downregulate the NF-κB axis, mitigating inflammatory injury induced by MIRI

**Fig. 6:**
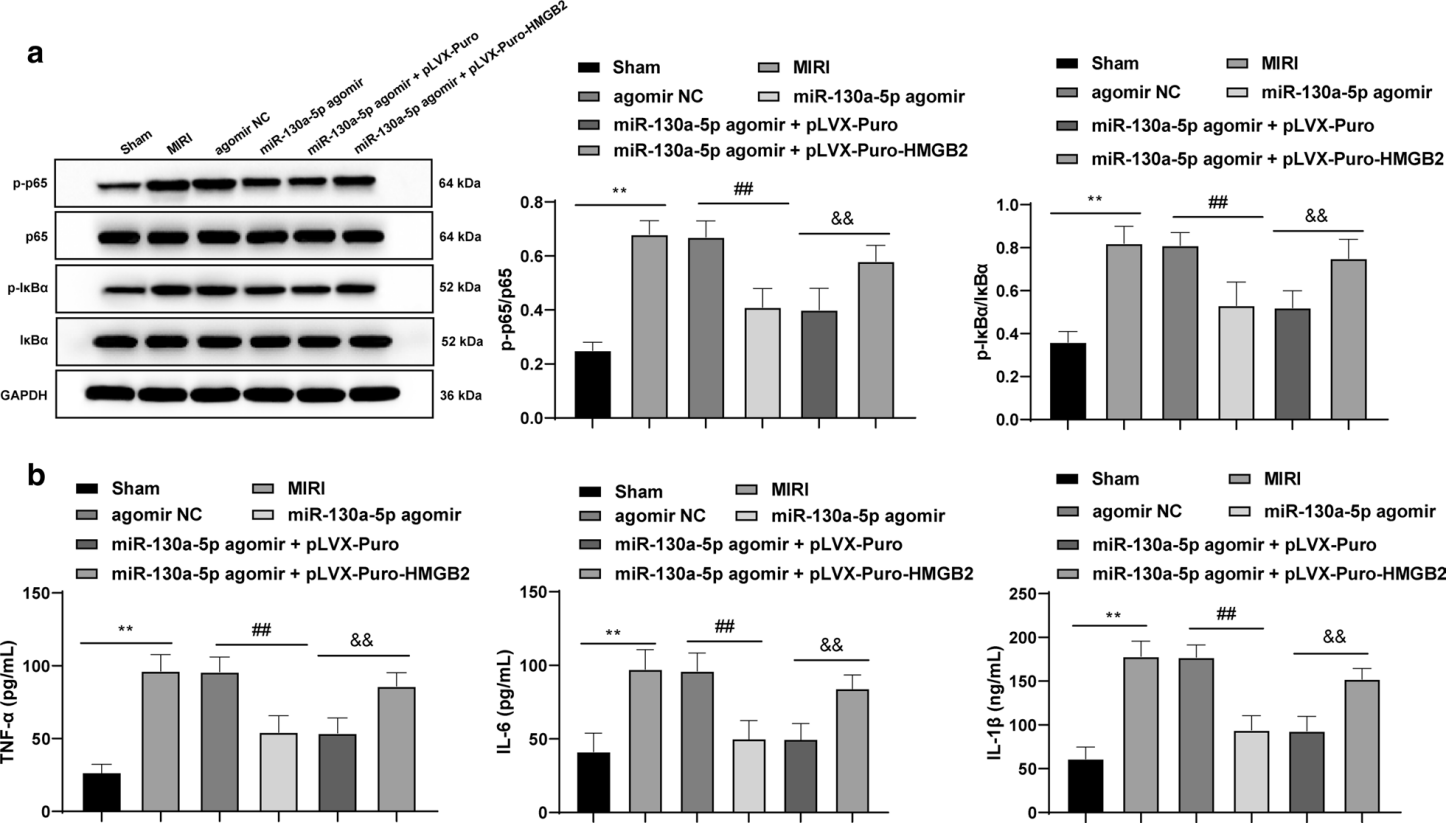
miR-130a-5p targets HMGB2 to downregulate the NF-κB axis, mitigating inflammatory injury induced by MIRI. **a** Western blot analysis was applied to assess the p65 and IκBα protein levels and phosphorylation in myocardial tissues in MIRI mice, n = 4. **b** ELISA kits were utilized to determine the serum levels of TNF-α, IL-6 and IL-1β, n = 10. The images of **a** shown are cropped. One-way ANOVA and Tukey’s multiple comparisons test were applied to determine statistical significance. **p < 0.01; ^##^*p* < 0.01; ^&&^*p* < 0.01. *miR* microRNA, *HMGB* high mobility group box, *NF-κB* nuclear factor kappa-B, *MIRI* myocardial ischemia reperfusion injury, *IκB* inhibitor of κB, *ELISA* enzyme-linked immunosorbent assay, *TNF-α* tumor necrosis factor-α, *IL* interleukin

In comparison with the sham-operated mice, in the MIRI mice, p65 and IκBα phosphorylation in the myocardial tissues was enhanced along with elevated serum levels of TNF-α, IL-6 and IL-1β. Overexpressing miR-130a-5p devoted to inverted results. However, upon up-regulation of HMGB2 in the MIRI mice with overexpressed miR-130a-5p, p65 and IκBα phosphorylation in the myocardial tissues was enhanced and the serum levels of TNF-α, IL-6 and IL-1β were also elevated correspondingly (all *p* < 0.01) (Fig. 6a, b).

## Discussion

The miR-130a expression was lower in the OGDR-treated cells compared to the normoxic cells [9]. Among the vast medical literature about MIRI, however, theme related to miR-130a-5p was seldom found, so we aimed to elucidate some potential MIRI therapies on the basis of miR-130a-5p. Consequently, our data showed that overexpressed miR-130a-5p suppressed MIRI by down-regulating the HMGB2/NF-κB axis.

Firstly, our results demonstrated significant decreases of EF, FS, LVSP and ± dp/dtmax along with a notable increase of LVEDP. As a diagnostic index of MIRI injury, decreased LVDP and ± dp/dtmax inferred a wide range of MI [19]. Then, our findings exhibited elevated CK and cTnT levels in the MIRI mice. As a pivotal diagnostic biomarker in MIRI, the activation of cTnT could have a detrimental impact on heart function [20]. CK and cTnT as common targets in heart disease were correlated with several cardiac specific miRs for regulating acute myocardial ischemia [21]. Consistently, these findings served as basis for a speculation supporting that miR-130-5p could downregulate CK and cTnT to subsequently alleviate MIRI progression. Also, MIRI mice presented with a high apoptosis rate, where Bax was up-regulated while Bcl-2 was down-regulated. Lower Bcl-2 level and higher Bax level were correspondingly evident in renal tissue subjected to IRI relative to the normal renal tissues [22]. In cerebral IRI, miR-130a hindered cell apoptosis induced by OGDR [9]. Subsequent gain-of assays suggested that overexpressed miR-130a-5p ameliorated MIRI by contributing to an impeded cardiomyocyte apoptosis and repairing the MIRI structural arrangement. Additionally, miR-130a, could help cells maintain homeostasis under hypoxic conditions induced due to oxidative stress, therefore alleviating peripheral artery disease [23]. Besides, overexpressed miR-130a-5p contributed to ameliorate oxidative stress and mitochondrial disorder consequent of MIRI. Additionally, miRs extensively modulated the mitochondrial activities, the chief organelle for cell apoptosis after MIRI [7]. miR-130a exerted protective effects against cerebral IRI [11]. The regulation of miR-130a-3p on mitochondrial functions was a potential development in fighting against cell senescence [24]. The aforementioned results discernibly indicated that miR-130a-5p relieved MIRI to a significant degree.

A prior study identified the oncogene HMGB2 as a downstream target of miR-130a [25]. Additionally, the results from dual-luciferase reporter gene assay ascertained the targeting relation between miR-130a-5p and HMGB2. In the glioma cells, miR-130a-5p overexpression exercised repressive effects by targeting HMGB2 [26]. Furthermore, our findings demonstrated that overexpressed HMGB2 could avert the protective effects of miR-130a-5p on MIRI mice. After IR, HMGB2 was elevated in the cardiomyocytes after MIRI, which enhanced the cell apoptosis, and led to increased Bax and decreased Bcl-2 levels [27]. Increased serum HMGB2 levels were associated with MI severity and deleterious cardiac events; hence the increased HMGB2 levels amplified myocardial ischemic injury in rats and hypoxic H9C2 cell damage via ROS induced by progressive glycation end products [28]. In hepatic IR, where HMBG1 was activated, the MDA level was also enhanced, conferring to a positive association between HMGB and oxidative stress [14]. As the major source of ROS and a vital mediator in the apoptotic process, mitochondria combined with the inflammation-triggered HMBG1to further exacerbate IRI [29]. Moreover, our findings revealed that miR-130a-5p targeted HMGB2 to downregulate the NF-κB axis so as to mitigate the inflammatory injury induced by MIRI. The involvement of NF-κB axis has been in reported in hepatic IR injury and renal I/R injury [15, 30]. HMGB2 silencing significantly inhibited I/R-induced cell proliferation reduction, cell apoptosis, activation of NF-κBp65 [16]. HMGB1 also aggravated myocardial I/R injury via regulation of the JNK1/2 and NF-κB pathway [31]. Captivatingly, slowdown of HMGB2 resulted in a reduced expression pattern of NF-κB in the cardiomyocytes under MIRI, which inferred the positive correlation between HMGB2 and NF-κB [27]. In ischemic stroke, manifestation of downregulated NF-κB was supplemented by the knockdown of TNF-α, IL-6 and IL-1β [32]. A recent study suggested that miR-130a targeted several pro-inflammatory cytokines to reduce the production of TNF-α, IL-6 and IL-12 [33]. All in all, miR-130a-5p was an attractive biomarker for relieving MIRI.

## Conclusion

To conclude, our study supported that overexpressed miR-130a-5p alleviated MIRI by downregulating the HMGB2/NF-κB axis. These results elicited a novel approach for the development of new MIRI treatment. Our future studies will focus at exploring the underlying mechanism of other targets of miR-130a-5p and to identify reliable therapeutic targets for MIRI. As this is a preclinical research, our findings provide therapeutic implication in MIRI treatment, and however our experiment results and effective application into clinical practice need further validation.

## Data Availability

The datasets used and/or analysed during the current study available from the corresponding author on reasonable request.

## Abbreviations

AIF: Apoptosis inducing factor
ANOVA: Analysis of variance
ATP: Adenosine triphosphate
Bax: Bcl2-Associated X
CK: Creatine kinase
cTnT: T Cardiac troponin T
Cyt-c: Cytochrome C
± dp/dtmax: Maximal rate of rise and fall
EDP: End-diastolic pressure
EF: Ejection fraction
FS: Fraction shortening
GAPDH: Glyceraldehyde-3-phosphate dehydrogenase
HE: Hematoxylin and eosin
HMGB: High mobility group box
IL: Interleukin
IκB: Inhibitor of Κb
LDH: Lactate dehydrogenase
LV: Left ventricle
MDA: Malondialdehyde
MI: Myocardial infarction
miR: MircoRNA
MIRI: Myocardial ischemia reperfusion injury
MUT: Mutant type
NC: Negative control
NF-κB: Nuclear factor kappa-B
OGDR: Oxygen–glucose deprivation reperfusion
ROS: Reactive oxygen species
RT-qPCR: Reverse transcription quantitative polymerase chain reaction
SOD: Superoxide dismutase
SP: Systolic pressure
TNF-α: Tumor necrosis factor-α
TUNEL: TdT-mediated dUTP nick-end labeling
WT: Wild type
